# Heme and menaquinone induced electron transport in lactic acid bacteria

**DOI:** 10.1186/1475-2859-8-28

**Published:** 2009-05-29

**Authors:** Rob Brooijmans, Bart Smit, Filipe Santos, Jan van Riel, Willem M de Vos, Jeroen Hugenholtz

**Affiliations:** 1 TI food & Nutrition, Kluyver Centre for Genomics of Industrial Fermentation, Po Box 557, 6700 AN, Wageningen, the Netherlands; 2 Wageningen University and Research Centre, Laboratory of Microbiology, Dreijenplein 10, Building 316, 6703 HB, Wageningen, the Netherlands; 3 Campina Innovation, Nieuwe Kanaal 7C, 6709PA, Wageningen, the Netherlands; 4 NIZO food research, PO Box 20, 6710 BA Ede, the Netherlands

## Abstract

**Background:**

For some lactic acid bacteria higher biomass production as a result of aerobic respiration has been reported upon supplementation with heme and menaquinone. In this report, we have studied a large number of species among lactic acid bacteria for the existence of this trait.

**Results:**

Heme- (and menaquinone) stimulated aerobic growth was observed for several species and genera of lactic acid bacteria. These include *Lactobacillus plantarum, Lactobacillus rhamnosus, Lactobacilllus brevis, Lactobacillus paralimentarius, Streptococcus entericus *and *Lactococcus garviae*. The increased biomass production without further acidification, which are respiration associated traits, are suitable for high-throughput screening as demonstrated by the screening of 8000 *Lactococcus lactis *insertion mutants. Respiration-negative insertion-mutants were found with *noxA*, *bd*-type cytochrome and menaquinol biosynthesis gene-disruptions. Phenotypic screening and *in silico *genome analysis suggest that respiration can be considered characteristic for certain species.

**Conclusion:**

We propose that the *cyd*-genes were present in the common ancestor of lactic acid bacteria, and that multiple gene-loss events best explains the observed distribution of these genes among the species.

## Background

Lactic acid bacteria are extensively used for the production of a diverse range of fermented foods with improved shelf-life, taste and nutritional properties [1-3]. The consumption of certain strains of lactic acid bacteria, called probiotics, may even provide health benefits by preventing or reducing disease symptoms [4,5]. Lactic acid bacteria are typically cultivated in (micro)anaerobic food-environments and have been (historically) classified as non-respiring, facultative anaerobes.

Since the early seventies, however, observations were made that heme could induce behavior that resembles respiration in several lactic acid bacteria that included *Lactococcus lactis*, *Enterococcus faecalis*, *Streptococcus *species and *Leuconostoc mesenteriodes*. Heme stimulated the aerobic growth of these species and/or induced cytochrome formation [6-9]. Recent experimental work provided conclusive evidence for actual respiration in *Lactococcus lactis*. Typically, respiratory chains consist of dehydrogenases, a membrane integral electron shuttle, such as quinones, and cytochromes and can generate a proton motive force. Menaquinone production by *Lactococcus lactis strains *has been observed, as well as, genes found that encode menaquinone biosynthesis in the sequenced genomes [10-12]. Moreover, the *Lactococcus lactis *respiratory chain contains a heme-dependent *bd*-type cytochrome, encoded by the *cydABCD *operon that is capable of generating a proton motive force [13,14].

Heme-induced respiration dramatically alters the phenotype of *Lactococcus lactis*, as it not only improves growth-efficiency but also robustness (improved stress resistance) [15,16]. The industrial relevance of these respiration-associated traits are made apparent by existing industrial and patent applications, for improved production of starter cultures [17,18].

Over the years several additional lactic acid bacteria were identified with a similar response to heme as *Lactococcus lactis*. A more structured investigation of the distribution of this trait among lactic acid bacteria, however, has not been conducted [19,20]. In this study, we aim to find more species of lactic acid bacteria that are potential respirators. As it also remains unclear whether respiration can be considered a species-specific trait, for a number of species multiple strains are examined. Furthermore, with the availability of numerous sequenced genomes, we can use *in silico *data as well to find potential respirators among the lactic acid bacteria.

## Methods

### Bacterial strains and growth conditions

For a full list of *Lactococcus lactis *and *Lactobacillus plantarum *strains used in this study see Table 1 and Additional File 1. The names of lactic acid bacteria, whose heme induced phenotypes are derived from other literature sources, are not included in Table 1, nor in the Additional File 1. Growth medium (MRS-broth or GM17-broth Merck, Amsterdam, the Netherlands [21]) was supplemented, when indicated, with heme (hemin) (Sigma, stock solution: 0.5 mg/ml in 0.05 M NaOH) to a final conc. of 2 μg/ml and/or vitamin K_2 _(menaquinone-4) (Sigma, stock solution: 2 mg/ml in ethanol) to a final conc. of 20 ng/ml. As a control the equivalent volume of 0.05 M NaOH or ethanol were added if no heme or menaquinone was added respectively.

**Table 1:** Lactic acid bacterial species and strains used in this study.

**Lactic acid bacteria used in this study**			
LAB species	NCC/other	LAB species	NCC/other
*Carnobacterium divergens*	DSM 20589	*Lactobacillus brevis*	B306
*Carnobacterium maltaromaticum*	DSM 20344	*Lactobacillus rhamnosus*	B637
*Carnobacterium gallinarum*	DSM 4847	*Lactobacillus delbrueckii delbrueckii*	B1799
*Enterococcus casseliflavus*	DSM 4841	*Lactobacillus gasseri VP*	B872
*Enterococcus faecalis*	B145	*Lactobacillus graminis*	B1356
*Enterococcus faecium*	B921	*Leuconstoc mesenteroides*	ATCC 8239
*Enterococcus flavescens*	DSM 7371	*Pediococcus pentosaceus*	DSM 20333
*Enterococcus mundii collins*	B919	*Pediococcus acidilactici*	B1697
*Lactococcus garviae*	DSM 6783	*Streptococcus entericus*	DSM 14446
*Lactococcus rafinolactis*	DSM 20443	*Streptococcus uberis*	B1118
*Lactobacillus sakei sakei*	23K	*Vagococcus fluvialis*	B102
*Lactobacillus paralimentarius*	B1357	*Weissella cibaria*	DSM 14295
*Lactobacillus garviae*	DSM 6783	*Weissella halotolerans*	DSM 20190
*Lactobacillus coryniformis torguens*	B284		

NCC: NIZO Culture Collection.

Aerobic growth conditions were achieved in shake flask cultivations with a 1:10 medium/volume ratio, while shaking at 250 rpm. For high throughput (96-wells micro-titer plates) aerobic cultivations, microtiter plates were filled with 150 μl medium/well (well volume μl 320), covered with breathseals and incubated in a microtron, shaking at 1000 rpm (Greiner Bio-one, Germany). The conditions of cultures grown in stationary tubes were considered anaerobic (or micro-aerobic). All strains were grown for 48 hours at 30°C before measuring biomass (optical density at 600 nm) and acidification.

### Measurement of menaquinone-content of bacterial cells

The following standard method was used for menaquinone measurement in cells; Overnight cultures were washed twice in phosphate buffer (50 mM K_2_HPO_4_, pH 5.0) and re-suspended to an OD600 of 10. Of this cell-suspension 2 ml was lysed by bead beating, using 0.1 mm silica-beads (Biospec products, Inc) in a Savant Bio 101 FastPrep FP120 and frozen till further use. Menaquinones were extracted by thoroughly mixing 500 μl of this lysed cell-suspension with 5 ml extraction buffer (90%hexane, 10% ethanol). The hexane layer was transferred to a new tube after centrifugation. This extraction procedure was repeated twice, and the combined hexane layers subsequently evaporated under nitrogen-gas. The precipitated menaquinones were re-dissolved in 300 μl ethanol. This menaquinone solution was analysed on a Thermo Finnigan (Waltham, MA) TSQ Quantum ms-ms system in combination with a Shimadzu (Kyoto, Japan) LC system. The samples were injected on a Synergi 4μ MAX-RP 80A 150 × 2 mm (Phenomenex) column where the compounds were eluted with a linear gradient, starting with 100% water/methanol 25/75 to 100% 2-propanol and detected with ms-ms. The TSQ Quantum ms system was equipped with an APCI (Atmospheric Pressure Chemical Ionization) source, set in the negative mode. A capillary temperature of 210°C was used with a vaporizer temperature of 300°C and a sheat gas pressure of 21psi. The collision energy for measuring in ms2 was set at 38 Volt for all compounds.

### Analysis of genomic content of sequenced lactic acid bacteria

The presence of the *cydABCD *and the menaquinone biosynthesis genes were based on annotation of the respective complete genome sequences and by homology analysis with *Lactococcus lactis *MG1363 gene-products, using BLASTP 2.2.18 (basic local alignment search tool) [12,22]. Comparison of genomic local organization was performed using the KEGG genome map , and the pinned region function of ERGO .

### DNA handling techniques

The identification of the genomic site of integration of the transposase gene, of the selected respiratory-defective, *Lactococcus lactis *B1157 mutants, was performed as described previously [23].

### Phylogenetic analysis of the *cyd*-genes

Each individual *cydABCD *gene product of *Lactococcus lactis *MG1363 was entered as a query to search for homologues in other lactic acid bacteria using the BLAST algorithm [22]. Sequence entries found to be homologous were retrieved (june '08) from GenBank, and separately aligned using the MUSCLE algorithm [24]. From the amino acid sequence alignments, bootstrapped neighbor joining trees were obtained using Clustal [25] with default settings, except for the number of iterations, which was set to 1000. Trees were analyzed in LOFT [26] and visualized in MEGA3 [27]. An identical exercise was carried out for 16S rRNA sequences from the organisms that were found to contain *cyd*-genes. Finally, the topology of the four trees, based on the *cyd*-gene products, was analyzed and compared to a standard phylogeny tree based on 16S rRNA.

## Results

### Screening for respiration in lactic acid bacteria

**Figure 1:**
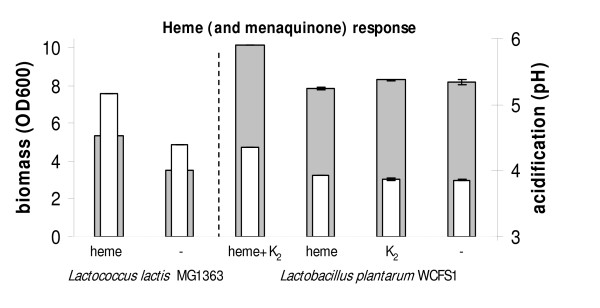
***Lactococcus lactis *MG1363 and *Lactobacillus plantarum *WCFS1 were grown aerobically overnight at 30°C in M17-medium and MRS-broth respectively**. A large biomass increase (grey bars), without further acidification (white bars), is observed when *Lactococcus lactis *is grown with heme, while *Lactobacillus plantarum *requires menaquinone (K_2_) in addition to heme. The error bars represent the standard deviation.

Respiration in *Lactococcus lactis *is easily induced by addition of heme, and results in enhanced growth efficiency with an almost doubling of biomass (Fig 1). Although *Lactococcus Lactis *cells grown without heme are less robust, in the complex medium used, extensive cell-lysis after the onset of the stationary phase is not apparent [15]. The final pH, at the end of the exponential growth phase tends to be higher in respiring cultures when compared to aerobic cultures (grown without heme). This higher final pH is a result of more progressive conversion of lactate to acetate during respiration [15]. We screened a diverse range of lactic acid bacteria (both species and strains) for similar heme induced growth stimulation. Of the 29 different species of lactic acid bacteria that were aerobically cultivated, 6 were clearly stimulated by the presence of heme (Table 2 and 3). In addition to *Lactococcus lactis*, the presence of heme led to increased biomass formation in *Carnobacterium divergens*, *Enterococcus casseliflavus*, *Lactobacillus paralimentarius*, *Streptococcus entericus*, and *Streptococcus uberis*, although the pH did drop.

**Table 2:** Heme-stimulated growth of lactic acid bacteria

Species		-		+heme	
			
		OD600	pH	OD600	pH
*Lactococcus lactis*	MG1363	3.52	4.39	5.36	5.16
*Enterococcus casseliflavus*	DSM 4841	1.13	6.09	3.56	4.83
*Streptococcus uberis*	B1118	0.91	5.93	2.00	5.17
*Carnobacterium divergens*^1^	DSM 20589	0.48	6.49	2.25	5.48

^1^has a high heme-dependent catalase activity

Lactic acid bacteria were grown aerobically in the presence (+heme) or absence (-) of heme (2 μg/ml). A clear increase in biomass yield is visible after a 48 hour incubation period. The experiments were performed at least in four-fold and the data shown represent averages.

**Table 3:** Heme and menaquinone-stimulated growth of lactic acid bacteria.

Species		+K_2_		+Heme		+heme +K_2_	
				
		OD600	pH	OD600	pH	OD600	pH
*Enterococcus faecalis*	B145	5.27	5.62	5.56	5.82	7.30	5.82
*Lactobacillus plantarum*	WCFS1	8.31	3.88	7.88	3.92	10.2	4.36
*Lactobacillus rhamnosus*	B637	1.55	4.15	1.51	4.17	3.59	4.01
*Lactococcus garviae*	DSM 6783	2.84	4.61	2.98	4.77	3.57	4.80
*Lactobacillus brevis*	B306	8.43	4.15	9.05	4.14	10.5	4.17
*Lactobacillus paralimentarius*^1^	B1357	1.17	4.51	2.21	4.28	1.98	4.37
*Streptococcus entericus*^1^	B2339	3.96	4.42	5.1	5.48	5.32	5.49
		g/l		g/l		g/l	
*Streptococcus agalactiae*^2^	NEM316	1.4	4.8	1.4	4.8	2.1	5.6

^1^Should be considered heme-stimulated ^2^see reference [19]

Lactic acid bacteria were grown aerobically in the presence of 2 μg/ml heme (+heme) and/or 20 μg/ml vitamin K_2 _(+K_2_), or with equivalent volumes of ethanol or 0.05 M NaOH as control. A clear increase in biomass yield is visible after a 48 hour incubation period when both heme and vitamin K_2 _are added to the growth medium. The experiments were performed at least in four-fold and the data shown represents averages.

Menaquinones form a part of electron transport chains, facilitating membranous electron transfer [28]. Many lactic acid bacteria are unable to produce menaquinones (more widely known as vitamin K_2_). Addition of both heme and menaquinone to aerated cultures of *Lactobacillus plantarum *WCFS1 increased the final (stationary phase) biomass and pH, quite similar to the heme supplemented phenotype of *Lactococcus lactis *(Fig 1 and Table 3). This is in agreement with the absence of a complete gene-set, in *Lactobacillus plantarum*, for menaquinone biosynthesis . The measured biomass (OD600) levels of *Lactobacillus plantarum*, grown with or without heme and menaquinone, remained relatively stable after the onset of the stationary phase (data not shown). In our selection of lactic acid bacteria we observed that *Lactococcus garviae*, *Lactobacillus rhamnosus*, *Lactobacillus brevis *and *Enterococcus faecalis *also require both heme and menaquinone for aerobic growth stimulation (Table 3). As reported in literature, a combination of these cofactors also stimulate biomass formation in *Streptococcus agalactiae *NEM316 [20]. Furthermore, heme-induced cytochrome formation in *Leuconostoc mesenteriodes *(NCIB 9917) and *Enterococcus faecalis *(V538) has also been reported [6,19,29,30].

### Distribution of *cyd*-genes in lactic acid bacteria

Currently there are 62 sequenced genomes of lactic acid bacteria available in the NCBI database of which 45 are complete . We examined the potential for respiration in this diverse set of lactic acid bacteria as indicated by the presence of the *cydABCD *genes in their genomes. The *cydA *and *cydB *encode for the structural subunits, and *cydC *and *cydD *are required for assembly of the *bd*-type cytochrome [31]. Using BLASTP and available annotation, we found that in 22 of these genomes all four *cyd*-genes were present (Table 4) [22].

**Table 4:** The presence of cytochrome genes in the sequenced lactic acid bacteria.

locus annotated as *bd-*cytochrome genes or with BLAST similarity%^a^:				
	structural subunits I & II		associated ABC transporter	
Species	(*cydA*)	(*cydB*)	(*cydC*)	(*cydD*)

*Enterococcus faecalis *V583	EF2061	EF2060	EF2059^e^	EF2058
*Lactobacillus brevis *ATCC 367	LVIS_1642	LVIS_1641		LVIS_1639
*Lactobacillus casei *ATCC 334	LSEI_2205 LSEI_0012^b^	LSEI_2204	LSEI_2203	LSEI_2202
*Lactobacillus gasseri *ATCC 33323	LGAS_1841	LGAS_1842	LGAS_1843	LGAS_1844
*Lactobacillus johnsonii *NCC 533	LJ1810	LJ1811	LJ1812 (2e-116)	LJ1813 (5e-110)
*Lactobacillus plantarum *WCFS1	lp_1125	lp_1126	lp_1128	lp_1129
*Lactobacillus reuteri *100-23	^e^Lr_0600	^e^Lr_0599	^e^Lr_0598 (3e-140)	^e^Lr_0597(3e-120)
*Lactobacillus reuteri *F275	Lreu_0505	Lreu_0506	Lreu_0507 (1e-136)	Lreu_0508 (6e-121)
*Lactobacillus salivarius *UCC118	LSL_1032	LSL_1031	LSL_1030 (1e-137)	LSL_1029 (3e-131)
*Lactococcus lactis *MG1363	llmg_1864	llmg_1863	llmg_1862	llmg_1861
*Lactococcus lactis *SK11	lacr_0737	lacr_0738	lacr_0739	lacr_0740
*Lactococcus lactis *Il1403	L107762	L109201	L110479	L112352
*Leuconostoc mesenteroides *ATCC 8293	LEUM_0560	LEUM_0561	LEUM_0562^d^	LEUM_0563^c^
*Oenococcus oeni *ATCC BAA-1163	OENOO_65065	OENOO_65064	OENOO_66040	OENOO_66039
*Oenococcus oeni *PSU-1	OEOE_1837	OEOE_1836	OEOE_0414	OEOE_0415
*Streptococcus agalactiae *18RS21	SAJ_1696	SAJ_1694	SAJ_1693^d^	SAJ_1692^c^
*Streptococcus agalactiae *2603V/R	SAG1742	SAG1741	SAG1739^d^	SAG1740^c^
*Streptococcus agalactiae *515	SAL_1843	SAL_1842	SAL_1841^d^	SAL_1840^c^
*Streptococcus agalactiae *A909	SAK_1750	SAK_1749	SAK_1748^d^	SAK_1747^c^
*Streptococcus agalactiae *CJB111	SAM_1705	SAM_1704	SAM_1703^d^	SAM_1702^c^
*Streptococcus agalactiae *COH1	SAN_1868	SAN_1867	SAN_1866^d^	SAN_1865^c^
*Streptococcus agalactiae *H36B	SAI_1857	SAI_1856	SAI_1855^d^	SAI_1851^c^
*Streptococcus agalactiae *NEM316	gbs1787 (2e-140)	gbs1786 (1e-99)	gbs1785 (1e-142)	gbs1784 (5e-132)

^a^Blast against MG1363 *cyd*-genes ^b^fusion of subunit 1 and subunit 2 ^c^Best hit with MG1363 CydC, annotated as CydD ^d^Best hit with MG1363 CydD, annotated as CydC ^e^LVIS_1640 is a pseudo gene SAJ_1695 is a cydA pseudo gene SAI_1865 E = 5e-37 annotated as cydA SAI_1866 E = 2e-7 annotated as cydA, ^e^Lr is an abbreviation of "Lreu23DRAFT".

All *cydABCD *genes were absent in *Lactobacillus sakei *23K, *Lactobacillus delbrueckii *(strains ATCC 11842, ATCC BAA-365), *Enterococcus faecium *DO, *Lactobacillus acidophilus *NCFM, *Pediococcus pentosaceus *ATCC 25745, *Streptococcus gordonii *CH1, *Streptococcus mutans *UA159, *Streptococcus pneumoniae *(strains D39, R6, SP11-BS70, SP14-BS69, SP18-BS74, SP19-BS75, SP23-BS72, SP3-BS71, SP6-BS73, SP9-BS68, TIGR4), *Streptococcus pyogenes *(strains M1 GAS, M49 591, MGAS10270, MGAS10394, MGAS10750, MGAS2096, MGAS315, MGAS5005, MGAS6180, MGAS8232, MGAS9429, SSI-1, Manfredo), *Streptococcus sanguinis *SK36, *Streptococcus suis *(strains 05ZYH33, 89/1591, 98HAH33) and *Streptococcus thermophilus *(strains CNRZ1066, LMD-9, LMG 18311).

In the case of *Lactobacillus brevis *ATCC367 *cydC *has degenerated to a pseudo gene. *Streptococcus agalactiae *NEM316 has four open reading frames (gbs1787-1784) with a high similarity to *cydABCD *of *Lactococcus lactis *MG1363 and a similar operon structure. *Lactobacillus casei *ATCC334 possesses, besides a *cydABCD*- operon, a separate fusion gene of *cydA *and *cydB*. The genome of *Streptococcus agalactiae *H36B contains 3 genes that are annotated as *cydA *Of these, SAI_1857 lies in an operon together with *cydBCD *while SAI_1865 and SAI_1866 are next to each other on the chromosome. SAI_1865 and SAI_1866 are truncated versions of *cydA *and are unlikely to encode a functional subunit I of the *bd*-type cytochrome.

Although, there is some confusion in the annotation of the open reading frames as either *cydC *or *cydD *genes among the lactic acid bacteria, the presence or absence of all four if the *cyd*-genes could be unambiguously determined for the studied sequenced genomes.

**Figure 2:**
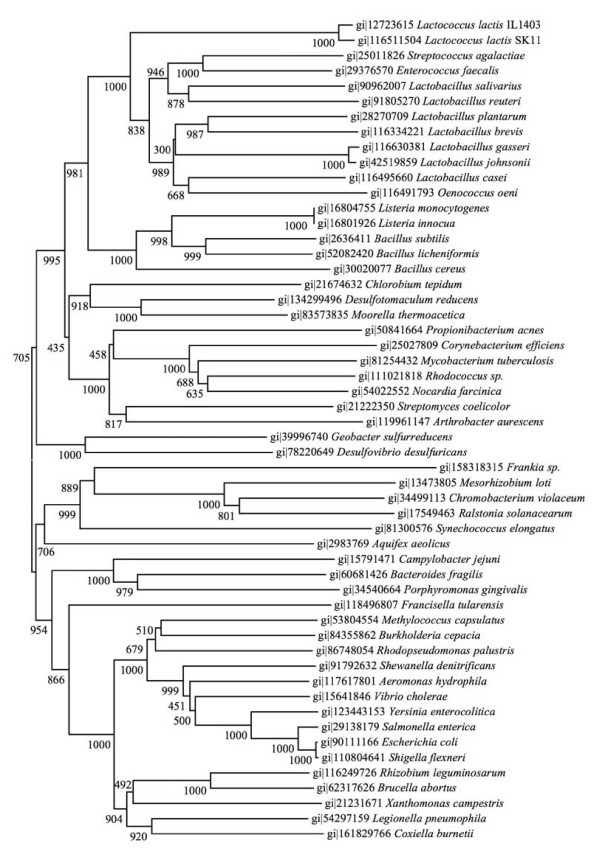
**Phylogenetic tree based on *cydA *sequences, constructed as explained in the materials and methods section**.

Next, we constructed a phylogenetic tree for each of the *cydABCD *genes. For this purpose, we used the *cyd*-gene products (AA-sequences) found in the lactic acid bacteria, and also as found in many non-lactic acid bacteria. Overall, the topologies of the phylogenetic trees made for *cydA*, *cydB*, *cydC *and *cydD*, resembled each other and the canonical phylogenetic (evolutionary) trees based on 16S rRNA. In all cases the lactic acid bacteria cluster together, and are neighbored by representatives of *Listeria *and *Bacillus*, forming the Firmicutes branch (Fig 2).

We compared the responses to heme (and menaquinone) (both observed in this work and reported in literature) with the genomic presence of the *cydABCD *genes and menaquinone biosynthesis. For the sequenced species *Lactococcus lactis *MG1363, SK11, *Lactobacillus plantarum *WCFS1, *Enterococcus faecalis *V583, *Streptococcus agalactiae *NEM316 there is a match between genotype and phenotype. Heme enhanced aerobic growth (biomass) has also been observed for *Oenococcus oeni *(A. Gruss presented at the LAB9 congress, Egmond aan Zee, 31 Aug–4 Sep, 2008). These species show respiration behavior (either biomass stimulation or formation of cytochromes) and have *cyd*-genes present on their genomes. Furthermore, as mentioned, *Lactococcus lactis *is known to produces menaquinones, whereas both *Lactobacillus plantarum *WCFS1 and *Streptococcus agalactiae *NEM316 lack many genes typically involved in menaquinone biosynthesis . This is thus also in line with our observations of the dependency on both heme and menaquinone. Also relevant, in this respect is that we did not observe that heme stimulated biomass formation in *Lactobacillus sakei *23K, *Lactobacillus delbrueckii *B1799 (not sequenced) or in *Pediococcus pentosaceus *DSM 20333.

There are some discrepancies however, as the sequenced *Leuconostoc mesenteroides *ATCC 8239 has all four *cyd*-genes and menaquinone biosynthesis genes, but in our hands, did not show enhanced production of biomass, in the presence of heme (and menaquinone). Heme-induced cytochrome formation has, however, been reported for other strains of *Leuconostoc mesenteroides *[6]. Furthermore, *Enterococcus faecalis *B145 required both heme and menaquinone, while the sequenced strain *Enterococcus faecalis *V538 appears to have a complete menaquinone biosynthesis pathway .

### Menaquinone production by *Lactococcus lactis *MG1363

The production of menaquinones by several lactic acid bacteria, such as *Lactococcus lactis *and *Leuconostoc mesenteroides *has been observed during non-respiratory (anaeorobic) conditions [10]. We have cultivated the *Lactococcus lactis *MG1363, in both respiratory and non-respiratory conditions to study its influence on the composition of the menaquinone pool (Table 5). Menaquinones can vary in their side-chain length (or the number of isoprenoid residues) although species with more than 10 isoprenoid residues are not typically found in (grampositive) lactic acid bacteria [32]. Assuming that the levels of menaquinone with 11 or more isoprenoid residues in their side-chain are negligible, we observed that, contrary to expectations, the total production of menaquinones was highest in anaerobic conditions by roughly 2-fold. In particular, the level of the menaquinone k_2_(3) was elevated in anaerobic conditions. In aerobic conditions, the concentration of menaquinone-species with 9 and 10 isoprenoid residues (k_2_(9), k_2_(10)) were elevated.

**Table 5:** Menaquinone (K_2_) production by *Lactococcus lactis *MG1363.

	Menaquinone content (μg/L)									
	
growth condition	K_2_(2)	K_2_(3)	K_2_(4)	K_2_(5)	K_2_(6)	K_2_(7)	K_2_(8)	K_2_(9)	K_2_(10)	total
Heme O_2_	0.0	12.6	3.3	2.9	1.8	3.3	13.7	28.9	0.7	67.0
O_2_	0.0	7.1	2.2	2.2	2.5	5.4	16.4	26.8	0.5	63.2
Heme	0.5	74.6	3.5	3.5	2.4	3.2	8.1	12.1	0.1	113.3
-	0.6	77.6	3.9	3.9	3.5	6.9	18.4	21.4	0.2	142.0

*L. lactis *MG1363 was grown overnight with(out) 2 μg/ml heme and with(out) aeration. Washed cells were analyzed for menaquinone content on HPLC. Menaquinone species with side-chain lengths varying from 2 to 10 (K_2_(2) - K_2_(10)) isoprenoid residues could be distinguished.

### Respiration is species-specific

The presence or absence of the *cyd*-genes in various species is remarkably consistent, as shown in table 4 and the corresponding text. The *cyd*-genes were not present in the (completely sequenced) genomes of any of the strains of *Streptococcus pyogenes *(13), *Streptococcus pneumoniae *(11), *Streptococcus suis *(2), *Streptococcus thermophilus *(3) and *Lactobacillus delbrueckii *(2), but consistently present in all *Streptococcus agalactiae *(8), *Lactococcus lactis *(3), *Lactobacillus reuteri *(2) and *Oenococcus oeni *(2) strains. Furthermore, in two separate studies 43 strains of *Lactobacillus plantarum *were genotyped and their genomic content compared to the genome of *Lactobacillus plantarum *WCFS1. In all these 43 genomes the *cydABCD *operon was determined to be present [33] (and Tseneva, personal communication). To determine if the respiratory-phenotype is as consistent as the genotype, we tested 88 strains of *Lactococcus lactis *(including SK11, MG1363 and IL1403) and 20 strains of *Lactobacillus plantarum *(including WCFS1). Heme (and vitamin K_2_) induced the respiratory response (enhanced biomass formation) in 95% of the *Lactococcus lactis *strains, and in 80% of the *Lactobacillus plantarum *strains (data not shown). One of the 4 *Lactococcus lactis *strains that did not respond to heme was the sequenced strain IL1403. In this particular case, we observed that a transposase is situated directly in front of the *cyd*-genes on the genome, which could be responsible for this lack of heme-induced response. For the other 3 *Lactococcus lactis *strains, that did not respond to the presence of heme, no extensive genomic information is available. Both the genotypic data and the growth experiments indicate that respiration is characteristic for a diverse group of lactic acid bacterial species.

### High-throughput respiration screening

By using an insertion knock-out bank of *Lactococcus lactis *B1157, we show that high throughput screening, in 96-well plates, is possible to isolate heme-stimulated lactic acid bacteria [23]. Approximately 8000 insertion mutants were aerobically incubated (with or without heme) and screened on biomass yield.

A total of 73 mutants were found which showed no significant biomass increase upon cultivation with heme. The position of integration of the insertion-knockout vector was determined in 13 of these 74 mutants by analysis of the surrounding genome sequence. The genomic region and the genes of *Lactococcus lactis *B1157 that were disrupted by the insertion event were identified by comparison with the *Lactococcus lactis *MG1363 genome sequence (Table 6).

**Table 6:** Disrupted genes in the *Lactococcus lactis *B1157 respiration negative mutants.

Mutant	sequence length (bp)	match Length (bp)	Identities MG1363	Genomic features of disruption location	
				
				locus	annotation
737_11	939	114	100%	llmg_0196	Geranylgeranyl pyrophosphate synthase
1C-6E	750	569	97%	llmg_0197	menA, 4-hydroxybenzoate polyprenyltransferase and related prenyltransferases
6B-4D	650	649	98%	llmg_1315	putative RNA methyltransferase
7D-9C	800	421	99%	llmg_1607	hypothetical protein
				llmg_1608	putative glycosyl hydrolases
737_16	897	894	100%	llmg_1735	noxA, NADH dehydrogenase, FAD-containing subunit
11D-2E	800	547	99%	llmg_1830	menX, menaquinone biosynthesis related protein
734_24	700	569	99%	llmg_1832	menE, Acyl-CoA synthetases (AMP-forming)/AMP-acid ligases II
734_17	600	248	99%	llmg_1833	menC, o-succinylbenzoate synthase
734_27	800	155	99%	llmg_1833	menC, o-succinylbenzoate synthase
737_4	750	541	100%	llmg_1861	cydD, cytochrome D ABC transporter ATP binding and permease protein
734_18	450	450	91%	llmg_1861	cydD, cytochrome D ABC transporter ATP binding and permease protein
737_12	700	29	100%	llmg_1863	cydB, cytochrome d ubiquinol oxidase, subunit II
734_1	750	750	100%	llmg_1938	aroB, 3-dehydroquinate synthase
				llmg_1939	aroE, hikimate 5-dehydrogenase

The sequence length of the genomic region of insertion is shown and the match to known *Lactococcus lactis *MG1363 gene sequences that were present in this region.

Five of the isolated respiration mutants carried mutations in genes involved in menaquinone biosynthesis. Respiration mutants with disruptions of *aroB *and *aroE *are also likely to suffer from an impaired menaquinone biosynthesis, since these genes are involved in synthesis of chorismate, the menaquinone precursor molecule. Three mutants were found that carried disruptions in the *cyd*-genes that are obviously essential to synthesize the *bd*-type cytochrome. Furthermore, we have now experimental evidence that a disruption of *noxA*, annotated as a NADH-dehydrogenase is directly linked to a respiration negative phenotype. 11 out of 13 respiration mutants carry mutations in genes that can be readily explained and thus validate this high throughput method to screen for respiration in lactic acid bacteria.

## Discussion

*Lactococcus lactis *MG1363 is capable of true respiration, as shown by the formation of a proton motive force by its heme-dependent electron transport chain [13]. We have used the characteristic phenotype of respiring *Lactococcus lactis*, a higher biomass with less extensive acidification, to screen for other possibly respiring lactic acid bacteria. Besides increased biomass production, respiration includes other traits, such as enhanced robustness. This makes the observation, that certain lactic acid bacteria are respirators, relevant for industrial applications [15,16]. Induction respiratory behaviour requires supplementation with heme and menaquinone (vitamin K_2_) in several additional species of lactic acid bacteria.

In the many cases, the heme-stimulated species showed enhanced biomass formation without a higher final pH. In these cases, although heme enhances aerobic growth, possibly as a result of energy conservation and the protection against oxidative stress afforded by the electron transport chain, changes in type of acids produced seems less important.

The addition of heme and menaquinone to the growth medium, to induce respiration in several lactic acid bacteria, may appear contrived. However, plants, the natural source of isolation of many lactic acid bacteria, can provide both heme and menaquinone (A. Gruss presented at the LAB9 congress, Egmond aan Zee, 31 Aug–4 Sep, 2008, and personal communications).

The *cyd*-genes (*cydABCD*) are present in many species of lactic acid bacteria. Most sequenced lactic acid bacteria that were screened showed a match between their genotype (*cyd*-genes, menaquinone biosynthesis genes) and the heme (and menaquinone) induced phenotype. Also the dependence and independence of a menaquinone source, in for example the *Lactobacillus *species and *Lactococcus *species, can be explained for some cases, with the presence/absence of menaquinone biosynthesis.

The *cyd*-genes are not confined to a limited subset of (closely related) species of lactic acid bacteria. In fact, the *cyd*-genes are present in many species that together span the diversity-range found in lactic acid bacteria [34]. What is remarkable is that the *cyd*-genes are so consistently present (or absent) in all the (sequenced) strains of a certain species, as can be clearly seen for the members of the genus *Streptococcus*. This uniformity may be the result of a bias in the isolation of the strains from highly similar niches. We have, however, screened a large number of *Lactobacillus plantarum *and *Lactococcus lactis *strains that were isolated from a variety of both industrial and plant-sources (see the materials and methods section). In both cases the induction of respiration by addition of heme and menaquinone was highly uniform with only a few exceptions. We can conclude that respiration is characteristic trait for, at least, *Lactococcus lactis *and *Lactobacillus plantarum*.

The high throughput screening of 8000 insertion mutants of *Lactococcus lactis *revealed that a high proportion of the respiration-impaired mutants contained insertions in menaquinone-biosynthesis genes. This implies that such methods can be used to screen for menaquinone producers among lactic acid bacteria. Those lactic acid bacteria that are stimulated by heme alone are potential producers of menaquinones.

Roughly half of the sequenced lactic acid bacteria contain the *cydABCD *genes. We investigated whether such a distribution could best be explained by horizontal gene transfer or, alternatively, by gene loss. The phylogenetic tree, constructed with the *cyd *genes sequences (Fig 2) is highly similar to the canonical (16S rRNA) evolutionary tree. Thus all lactic acid bacteria group together in one separate branch, which indicates ancient origins of the *bd*-type cytochrome. The results presented here not only support the idea that the *cyd *genes were present in the common ancestor of lactic acid bacteria, but in fact of all Firmicutes. Thus gene loss events best explains the observed *cyd*-gene distribution amongst lactic acid bacteria, which is in line with their highly auxotrophic nature. Lactic acid bacteria as a group have a history of adaptation to nutrient rich food-environments and progressive gene-loss that was nicely visualised by Makarova et.al. [35]. A typical example of this process is the extensive gene decay (high abundance of pseudo-genes) in the yoghurt-bacterium *Streptococcus thermophilus *[36].

(Mena)quinones are best known as cofactors of bacterial respiratory chains, shuttling electrons from dehydrogenases to the terminal oxidase. Menaquinone production in anaerobic, non-heme supplemented conditions have been reported in literature before for other *Lactococcus lactis *strains and *Leuconostoc *sp. [10]. Several groups have proposed an additional role of menaquinones in offering protection against oxidative stress [37,38]. Recently it has been shown that quinones of *Lactococcus lactis *can reduce metal-ions such as Fe^3+ ^and Cu^2+^, which may facilitate their assimilation [39]. Still, contrary to expectations when *Lactococcus lactis *was grown aerobically (both with and without heme), the total amount of menaquinones produced was almost two-fold lower compared with anaerobic conditions. Furthermore we observed that aerobic cultivation induces an altered composition of the menaquinone pool, with a shift towards menaquinones with more 9–10 isoprenoid residues in their side-chain. This study reports that respiration in several lactic acid bacteria can be induced by a combination of heme and vitamin K_2_(4) (with four isoprenoid residues). It is thus not known what function the observed shift, in the composition of the menaquinone-pool to menaquinones with a longer side-chain length, serves in these bacteria. In humans, however, menaquinones with longer side-chain remain detectable for longer times in the blood stream and may form a more available source of vitamin K_2 _[40]. Therefore, induction of the production of menaquinone with longer side-chains by lactic acid bacteria may better fulfil human vitamin K_2 _requirements. The function of menaquinones, in the (anaerobic) metabolism of *Lactococcus lactis*, is unclear. For example, the various menaquinone mutants of *Lactococcus lactis *grew well anaerobically and aerobically (data not shown). In fact many species of lactic acid bacteria grow well both anaerobically as aerobically, although not all of these produce menaquinones. Since, lactic acid bacteria do not depend on menaquinone for growth they make ideal organisms to study the impact of the menaquinones, with various side-chain lengths, on (respiratory) metabolism.

This work has revealed that a number of known lactic acid bacteria are potential respirators which, as in the case of *Lactococcus lactis*, could be targeted for future industrial exploitation.

## Competing interests

The authors declare that they have no competing interests.

## Authors' contributions

RB did (almost) all of the experimental work and the writing. BS provided the knock-out library of *Lactococcus lactis*, FS provided essential input for the phylogeny trees, JR performed the menaquinone analysis in all bacteria, WV served as main advisor for most of the molecular biology work and JH acted as overall supervisor (and corresponding author) of the work. All authors have read and approved the final version of the manuscript.

## Supplementary Material

Additional file 1 ***Lactobacillus plantarum *and *Lactococcus lactis *strains used in this study**. A list of all *L. plantarum *and *L. lactis *strains used in this study. Click here for file
